# Does computer use pose a hazard for future long-term sickness absence?

**DOI:** 10.1186/1477-5751-9-1

**Published:** 2010-03-22

**Authors:** Johan H Andersen, Sigurd Mikkelsen

**Affiliations:** 1 Danish Ramazzini Centre, Department of Occupational Medicine, Herning Hospital, 7400 Herning, Denmark; 2 Department of Occupational Medicine, Copenhagen University Hospital, Glostrup, Denmark

## Abstract

The aim of the study was to investigate if weekly duration of computer use predicted sickness absence for more than two weeks at a later time.

A cohort of 2146 frequent computer users filled in a questionnaire at baseline and was followed for one year with continuously recording of the duration of computer use and furthermore followed for 300 weeks in a central register of sickness absence for more than 2 weeks.

147 participants of the 2,146 (6.9%) became first time sick listed in the follow-up period. Overall, mean weekly computer use did not turn out to be a risk factor for later sickness absence. The hazard ratio for sickness absence with weekly increase of one hour in computer use was 0.99 (95% CI: 0.99 to 1.00). Low satisfaction with work place arrangements and female gender both doubled the risk of sickness absence.

We have earlier found that computer use did not predict persistent pain in the neck and upper limb, and it seems that computer use neither predicts future long-term sickness absence of all causes.

## Findings

Computer use has for decades been associated with musculoskeletal pain problems, especially with acute pain, but less evidence exists on prolonged or chronic pain problems and consequences on sick leave. In general, computer professionals and technicians have been found to have a low risk of sickness absence [1], but this was based on a small sample of the Danish work force, and none of the studies, which have been performed among computer users, has analyzed the relation between computer use and sickness absence in detail. Recently, methodologies for assessing work activity during computer use has been evaluated, and activity-based recordings of computer use seem to be reliable and in agreement with measures obtained by observational techniques [2-4]. Two studies have used objective measures of computer activity, and found no association between computer use, mouse use or keyboard use in relation to the risk of persistent symptoms in the neck and upper limbs [5,6]. In this study we aimed at assessing if duration of computer use was associated with a later risk of prolonged sickness absence (e.g. sickness absence for more than 2 weeks) for all causes.

We used a computer programme (WorkPaceRecorder, Wellnomics LTD/ErgoDirect) to record usage patterns for computer usage, and summarized usage parameters into mean weekly hours of using the computer for one-year from 2000 to 2001 among 2146 technical assistants, which was a subgroup of the NUDATA-study [5,7-10]. For computer usage time an interval of 30 seconds between events of using the computer was used as criterion for usage, allowing for reading from the screen and thinking about the next input move to be included in the measure of computer usage time. In order to reduce a huge amount of data, we summed daily statistics to weekly values to form WPR computer time (hours per week (h/w)).

These data has afterwards been linked to a nationwide register of sickness absence for more than 2 weeks - the DREAM-database. The DREAM database includes information on all public transfer payments administered by Danish ministries, municipalities, and Statistics Denmark for all Danish citizens on a weekly basis since 1991, including granted sickness absence compensation since 1996 [11]. Each person was linked by the way of their unique personal registration number, obtaining a database with baseline information and objective recording of computer use for one-year. In addition the database included recordings of sickness absence for more than 2 weeks from the beginning of data collection (the day they filled a baseline questionnaire) and 300 weeks forward.

From a baseline questionnaire a series of covariates regarding physical, psychosocial and individual characteristics were included in the analyses. They have earlier been described in detail [5,7-10]. In short, these variables were individual factors: gender, age, type A behaviour, negative affectivity, private social support, medical diseases with potential to influence neck and upper limb pain status, and accidents involving injury of the neck or shoulder; psychosocial work environment factors: job demands, job control, social support, job satisfaction and time pressure at work; ergonomic factors: abnormal position of mouse or keyboard, lack of arm/wrist support, height of screen, and adjustable work desk and chair. To account for other aspects of the arrangement of the workplace, a "mixed" ergonomic/psychosocial variable (*"How satisfied are you with the overall arrangement of your work place?"*) with response alternatives *very satisfied, satisfied, neither satisfied or unsatisfied, unsatisfied, very unsatisfied, don't know *was included.

Seniority was assessed by the length of time participants had used a computer at work to the same extent as currently, divided into less than three years, four to seven years, eight to ten years, and more than 10 years.

Data were analyzed by complementary log-log (CLL) regression for interval-censored survival times, where the time variable for week number was introduced into the model as an indicator variable: The CLL model is a discrete analogue of the continuous proportional hazards model. The outcome was time to first appearance of a sickness absence period for more than 2 weeks, with computer time as the explaining variable. Only those weeks where the participant was at risk of being sick-listed were counted (e.g., if people were unemployed for some weeks, this would not count as time at risk). Those participants who emigrated, retired or died during the follow-up period were censored.

The effect of computer time was estimated as a linear effect and by categories of computer time. A standard set of potential confounders was used for all analyses and included 1) individual factors, 2) psychosocial work environment factors, 3) ergonomic factors and 4) job task seniority in years of working with a computer. All analyses were conducted in STATA version 10.0.

The mean computer use among the participants was 9.2 hours per week (SD = 6.3 hours per week). One hundred and forty seven participants of the 2,146 (6.9%) became first time sick listed in this period of 300 weeks. The average risk time per person (table 1) was similar for the different computer time groups indicating that the results on sickness absence could not be explained by other differences in public transfer payments between the exposure groups. The hazard ratio for sickness absence with weekly increase of one hour in computer use was 0.99 (95% CI: 0.99 to 1.00). Table 1 shows the hazard ratios for sickness absence divided in to categories based on weekly usage times. The risk estimates were all around 1 without any pattern of exposure response between computer use and risk of sickness absence. The results were similar when we divided computer use into its main elements: mouse use and keyboard use. There was no effect of years working with a computer. Women had more than a double risk for sickness absence compared with men HR = 2.6 (2.4-2.8). Other factors associated with sickness absence were earlier accidents in the arm-hand region (HR = 1.7(1.5-2.0)). BMI below 20 predicted sickness absence (HR = 2.6(2.3-3.0)), and for BMI > 30 the risk was 3.1 (2.8-3.4). There were some effects of psychosocial work place factors, (high job demands (HR = 1.6 (1.5-1.8)), low satisfaction with work place arrangements (HR = 2.1 (1.9-2.3)). Questions on different aspects of ergonomic factors e.g. screen position, keyboard and mouse position, adjustable table and chair did not have any effect on sickness absence.

**Table 1:** Risk estimates for sickness absence for more than 2 weeks in relation to mean weekly computer use

Computer use Hours/week	Number of Participants N = 2,146	Sickness absence > 2 weeks (%)	Mean number weeks at risk per person	Sickness absence HR (95% CI) Unadjusted	Sickness absence HR (95% CI) Adjusted*
0-<2.5	365	7.3	257	1.00	1.00
2.5-<5	230	6.0	261	0.91 (0.82-0.99)	1.07 (0.96-1.20)
5-<10	628	7.1	261	1.05 (0.98-1.13)	1.19 (1.10-1.30)
10-<15	540	6.9	260	1.09 (1.01-1.17)	1.09 (0.99-1.19)
15-<20	276	6.0	262	1.09 (0.99-1.19)	1.04 (0.93-1.16
> 20	107	6.3	263	1.08 (0.96-1.23)	0.94 (0.82-1.09)

*Hazard ratios with 95% confidence intervals obtained by complementary log- log regression for interval-censored survival times. Adjusted for individual characteristics, psychosocial work environment factors, ergonomic factors and computer work seniority (years).

This paper presents risk estimates for sickness absence for more than 2 weeks in a cohort of computer users. We found no risk of higher sickness absence in relation to higher weekly usage of the computer. Higher sickness absence in this cohort was associated with female gender, low and high BMI, earlier injuries in the upper extremity, high job demands and overall low satisfaction with the work place arrangements, despite no specific effects of ergonomic factors. This study benefits from a homogeneous population of technicians, and the group that only uses their computer to a small extent (from zero to 2.5 hours per week), and who was used as the reference group was not exposed to hazardous non-office exposures. They attend meetings, talk in telephone and do other office work. Computer use was objectively registered for one-year, and we cannot exclude the possibility that usage pattern could change in the follow-up period, which were 300 weeks. If we restricted the analyses to the year of exposure measurements or the following year the associations were no different than reported in this paper. Introducing variability values for weekly computer use into the model did not change the estimates. We have no knowledge of reasons for sickness absence. Sickness absence is a complex sociological phenomenon with occupational as well as non-occupational determinants. Musculoskeletal disorders and common mental health problems are among the most important factors in long-term sickness absence. The DREAM-register only contains information of granted sickness absence compensation whatever the background and diagnoses. We hypothesized that if there were strong adverse health effects of computer use, which have been claimed for several years, it would show up as later sickness absence. But, we acknowledge that an effect on musculoskeletal sickness absence could be diluted by measuring all-cause sickness absence. Another explanation for the negative association between computer use and sickness absence could be that ailments in the form of pain problems from neck and upper extremity do not hamper the ability to go to work and therefore does not cause sickness absence. A Dutch study found that among computer users with neck/shoulder symptoms or hand/arm symptoms loss in productivity derives from a decreased performance at work and not from sickness absence [12]. Findings from earlier studies support that pain in the neck and upper extremity among computer users is mostly acute and transient pain, and in studies with clinical examinations very few diagnoses could be obtained [7,9,10]. Two recent studies found no association between recorded computer use and persistent pain [5,6]. So, we feel confident in concluding that computer work probably is associated with pain problems now and then, but the risk of more persistent or chronic disorders is small, and as this study reveals, does not cause long-term sickness absence.

## Competing interests

The authors declare that they have no competing interests.

## Authors' contributions

JHA and SM conceived of the study, and participated in its design and coordination and helped to draft the manuscript. JHA and SM participated in the design of the study and JHA performed the statistical analysis. All authors read and approved the final manuscript.
